# Testing the bounds of compassion in young children

**DOI:** 10.1098/rsos.221448

**Published:** 2023-02-15

**Authors:** James N. Kirby, Kelly Kirkland, Matti Wilks, Mitchell Green, Porntida Tanjitpiyanond, Nafisa Chowdhury, Mark Nielsen

**Affiliations:** ^1^ School of Psychology, The University of Queensland, QLD 4072, Australia; ^2^ Melbourne School of Psychological Sciences, University of Melbourne, VIC 3010, Australia; ^3^ Department of Psychology, University of Edinburgh, Edinburgh EH8 9YL, UK; ^4^ Faculty of Humanities, University of Johannesburg, 2006, South Africa

**Keywords:** compassion, competition, children, cost, motivation

## Abstract

Extensive research shows that, under the right circumstances, children are highly prosocial. Extending an already published paradigm, we aimed here to determine what factors might facilitate and inhibit compassionate behaviour. Across five experiments (*N* = 285), we provide new insight into the bounds of 4- to 5-year-old children's compassionate behaviour. In the first three experiments, we varied cost of compassion by changing the reward (Study 1), using explicit instructions (Study 2) and ownership (Study 3). In the final two experiments, we varied the target of the compassionate behaviour, examining adults compared with puppet targets (Study 4), and whether the target was an in-group member (Study 5). We found strong evidence that cost reduces compassionate responding. By contrast, the recipient of compassion did not appear to influence responding: children were equally likely to help a human adult and a puppet, and an in-group member and neutral agent. These findings demonstrate that for young children, personal cost appears to be a greater inhibitor to compassionate responding than who compassion is directed toward.

## 1. Testing the bounds of compassion in young children

Compassion is a complex prosocial motive, defined as the sensitivity to suffering in self and others, with a commitment to alleviate or prevent it [1]. Compassion is strongly linked to helping and comforting behaviour, which has been examined extensively in developmental psychology. For example, seminal work by Warneken & Tomasello [2] found that in their second year of life children engage in altruistic helping so others can complete an action-based goal. Other researchers have examined children's motivations to comfort others. They generally find that children engage regularly in comforting behaviour, such as offering verbal or physical support to an experimenter experiencing a negative state [3]. However, the extent to which comforting behaviour is extended to a distressed target varies. For example, sometimes toddlers will ignore or show no response to other's distress [4], and children aged between 4.5 and 6 years of age are selective with who they assist, preferring friends and treating non-friends less well when making resource allocation decisions [5]. An experiment by Green *et al*. [6] aimed to understand how willing children were to help and comfort when they incurred personal cost. The authors found that, when the cost was high, a 4-year-old would almost never help a target (e.g. sacrifice a reward), even when that target was distressed. In our current paper, we aim to extend on this to explore the boundaries of compassion; that is, to understand the factors that may facilitate or inhibit compassionate behaviour in children.

### 1.1. What is compassion?

Compassion is triggered by exposure to distress and suffering, which brings a diverse range of contextually appropriate emotions and actions that drive us to engage in care and prosocial behaviour to alleviate the observed suffering [1]. Typically, compassion is directed towards interactions with kin or allies [7,8], but can be expanded to a broader range of entities [9], such as strangers (e.g. charity), other species (e.g. whales and primates) and even sentient beings not yet born (e.g. concern about climate change for future generations).

Gilbert [1] proposes an evolutionary account of compassion, suggesting that it is an important biosocial motive, often competing with other motives (such as harm avoidance, status seeking and competition), and as such there are facilitators and inhibitors that turn on and off the compassionate algorithm. In compassion, facilitators are factors that increase the likelihood of the compassionate algorithm being activated. As such a range of approaches can facilitate compassionate behaviour [10,11], like using self-reassuring/friendly inner voice tones [10], primes of safety and security [12], and compassion meditations [10] that can stimulate physiological mechanisms (e.g. the vagus and the parasympathetic system). Improving facilitators does not necessarily result in compassionate behaviour if there are inhibitors present [13]. For example, there is an inherent tendency to form groups and discriminate based on in- and out-group biases [14]. Group biases influence compassionate responding, whereby compassion can be turned on for an in-group member (e.g. family and friend) and be inhibited for an out-group member (e.g. stranger). In the fields of altruism, prosociality and empathy, other biases have been found to reduce helping behaviour, such as the bystander effect [15], personal cost to self [16] and competitive self-interest [17].

### 1.2. Developmental studies examining compassion

It has been well established that children engage in instrumental and costly helping behaviours and will frequently share resources to help others [18–20]. Many studies have also examined boundary conditions on children's helping; children are more likely to help in-groups than out-groups [21], and in some cases, children will even help when their peers do not [22]. However, these studies generally have not examined how children respond to targets who express explicit suffering [6]. Moreover, in many cases, the cost of helping is low, which facilitates compassionate responding [6].

Research also shows that children engage in sympathetic and empathic responding [4]. However, these studies have typically focused on physiological responses, such as pupil dilation [23], rather than a behavioural action [24]. In an important review on child prosocial behaviour, Dunfield [3] outlined an empirically grounded framework for differentiating forms of prosocial behaviour based on the varieties of negative states the child must identify and overcome. These include three specific prosocial behaviours: (i) helping (instrumental need), (ii) sharing (unmet material desire), and (iii) comforting (emotional distress). According to Dunfield's [3] model, these three prosocial behaviours are all underpinned by a motivation to see the negative state alleviated. The definition we have adopted for our work also conceptualizes compassion as a motivation to alleviate suffering [1], which is highly similar to these prosocial constructs of helping and comforting.

A range of studies have examined comforting behaviour in the context of more explicit emotional suffering (i.e. visibly upset individuals) [3,4,23,25]. These studies suggest that toddlers typically respond to another's distress by either seeking comfort in their parents, exclaiming sympathetic sentiments towards the distressed target, or even ignoring the distressed target [4,23]. While less frequent, some toddlers have been shown to exhibit prosocial behaviours in response to another's distress [25,26]. For example, Svetlova *et al.* [25] found that 18- and 30-month-olds would attempt to help reduce the distress of an experimenter by giving them various objects. However, this help was more likely when it was instrumental help (e.g. handing a clothespin to experimenter who dropped it) as opposed to emotional help (e.g. experimenter is crying and giving them a teddy bear would make them happy). In addition, children were less likely to help when it was costly (e.g. give their own objects compared with experimental objects) [25]. Trommsdorff *et al*. [27] exposed preschool-aged children to a distressed adult and found that prosocial behaviour toward the target (that involved personal cost) was positively related to sympathy and negatively related to self-focused distress. Finally, Green *et al*. [6] found that 4-year-old children would help a distressed puppet almost always when there was no personal cost to themselves; but when there was a personal cost helping rates dropped to floor levels.

Steinbeis [28] proposed that most prosociality is costly (in terms of time, effort and opportunities), and cost needs to be regulated for prosocial behaviour to occur. In relation to helping behaviour, regulation of emotions in response to another's distress is proposed as an important mechanism underpinning helpful behaviour [1]. To help an emotionally distressed target, children thus need a sufficiently mature prefrontal cortex to enable regulatory control and prosocial behaviour. This could explain why across developmental studies of differing ages of children we see considerable variation in prosocial helping, particularly when the experiments involve cost and emotionally distressed targets. In sum, a growing body of research has documented how children will act compassionately towards emotionally distressed targets [6,25] but this research has not, to date, yielded detail on what explanatory factors account for the drop in helping when costly.

### 1.3. The current study

Using an already published paradigm, and drawing from the adult literature, our aim is to determine what factors might facilitate greater compassionate behaviour in young children [6]. The five experiments presented here use the compassionate responding paradigm developed by Green *et al*. [6], and in these series of experiments we systematically vary cost and target. In the first three studies, we engage in a more nuanced exploration of cost, varying the reward (Study 1), the presence of explicit instructions (Study 2) and the ownership of resources (Study 3). Across all three studies, we predicted that personal cost would inhibit compassionate responding. In Studies 4 and 5, we examine how the target of the compassion varies responding towards adults versus puppets (Study 4) and in-group versus out-group members (Study 5). Here, we predict that having targets that were adults (versus puppets) and in-groups (versus out-groups) would facilitate compassionate responding.

## 2. General method

All studies were preregistered on the Open Science Framework (see https://osf.io/e78tb/?view_only=ff46e0f0a9894023b507af5a526afac6). All the experimental protocols used across the five studies were cleared and approved in accordance with the ethical review processes of the University of Queensland, and within the guidelines of the National Health and Medical Research Council's guidelines (#2019000651). All studies employed variations on the Green *et al*. [6] compassionate responding paradigm in which children played a series of games alongside a puppet. In each game, the puppet is unable to complete the game and the child can opt to help or not. In the original paradigm, Green *et al*. [6] varied whether the puppet was visibly distressed or not, and whether there was a personal cost to the child to help the puppet (i.e. they had to give up their resources so the puppet can complete the task). In the current studies, we replicated the high-cost and distressed condition from the Green *et al*. [6] study, where all helping behaviour is considered compassionate responding.

Prior to being introduced to the games, children picked their three favourite stickers from a large selection. These constituted the rewards for completing the tasks in all studies with the exception of the no reward condition in Study 1. Children played three games with three different puppets—Millie the Monkey, Ellie the Elephant and George the Giraffe (images of the puppets are included in the electronic supplementary material). Each puppet participated in one of the three tasks and was never repeatedly paired with one task due to the randomization of puppets and task order.

Three tasks were used across each of the five studies. These were the (i) sorting task (i.e. place a range of different coloured blocks onto their matching-coloured rods), (ii) marble task (i.e. place marbles onto a base which has nine small holes to fit the marbles), and (iii) puzzle task (i.e. place puzzle pieces in correct space on a board). Each task requires a participant to place objects together (see electronic supplementary materials for details of each task). Each task had two sets of equipment so that the child and puppet/adult could complete the tasks separately. In each scenario, the child had the correct number of pieces to finish the task, while the puppet/adult did not have enough pieces (missing two pieces) and hence was unable to finish the task unless the children acted compassionately and shared their pieces.

In each game the puppet/adult realized that they did not have sufficient pieces to finish the task and became distressed that they were unable to receive a sticker. There were three distress prompts, which increased in the emotional intensity of suffering shown, which is outlined in table 1. This allowed three opportunities for the child to help. If the child helped after the suffering was shown by the puppet, it was operationalized as compassionate behaviour. The experimenter ended the task as soon as the child helped, or after the three prompts if the child did not help. Full scripts for all prompts are provided in the electronic supplementary material.

**Table 1.** The three distress prompts used, which increase in emotional intensity.

	distress prompts
prompt 1: verbalized distress	‘Oh no … I don't have enough 〈appropriate to the task pieces〉 to finish the game. Now I'm not going to get a sticker … What am I going to do?’
prompt 2: Increased emotion	‘Oh no … The time is almost up and I can't finish the game. I really wanted a sticker but I'm not going to get one now. I'm missing pieces and can't finish the game!’
prompt 3: crying	‘Oh no … I'm not going to get a sticker now … I really wanted one. I'm so upset, I think I am going to cry. Time is about to finish, what can I do? I don't have enough pieces to finish the game!’

We also assessed children's perception of the emotion of the puppet for all studies. At the end of the task, children were asked to indicate how the puppet felt when they realized that they did not have enough pieces to complete the task (see electronic supplementary material).

All studies in this manuscript made minor variations off this initial design, as described below.

## 3. Study 1: I'll help you, but what's the cost?

In this study, we aimed to systematically explore how reward influences children's compassionate responding. Green *et al*. [6] gave children their stickers at the end of all three tasks. In this way, children may have been extrinsically rewarded by the stickers, decreasing their intrinsic motivation to help [29]. To explore this, we examined how an immediate reward (after each task) and no reward (although children still received stickers at the end of the entire experiment for their participation but they did not know this) would influence children's responding compared with the cost condition adapted from the Green *et al*. [6] study. Notably, the no reward condition tests children's propensity to help in the absence of external reward. By contrast, children in the immediate reward condition received their sticker during the task, while the (slow) puppet was still finishing. This meant that they could help without losing their reward, thereby allowing us to isolate the role of reward in this paradigm. We predicted that the removal of the cost (no reward and immediate reward) would facilitate compassionate responding by children. Additionally, Green *et al*. [6] observed that children who did not help either consoled or disengaged from the puppet. Therefore, this experiment also examined these behaviours as an exploratory addition to the study.

### 3.1. Method

All participants were recruited through local childcare centres or a database of parents who had previously agreed to participate in research at a local university. Participation took place either at the childcare centre or in a dedicated child-friendly testing space at the local university. Informed consent was provided by the parents for all participants. An online randomizer (https://www.randomizer.org/) was used to allocate child to condition. Once the session began, children were introduced to the puppet/adult and the experimenter explained the rules of the task. The task only began after children indicated that they understood the task. Upon completion of the task, children and caregivers were thanked and children who participated at the university were offered a reward as a thank you for participating. This procedure was the same for this and all other studies in this paper.

In this study, we adapted the cost condition from the original paradigm [6], where the child had insufficient pieces to help the puppet and would, thus, have to give up a reward to act compassionately. In addition to this baseline (cost), we included two additional conditions: one where children did not receive any stickers during the game (no reward) and one where children received a reward after each task instead of at the end of all three (reward immediate condition). Notably, when the puppet was distressed in the no reward condition, they did not refer to the sticker in their distress prompt—instead saying ‘Oh no … I don't have enough 〈task pieces〉 to finish the game. Now I am not going to be able to finish the game … what am I going to do?’.

#### 3.1.1. Participants

Sixty-eight children in total participated in this study. Eight children were excluded from the final analysis due to participant error (*n* = 1), experimenter error (*n* = 6) or failing to engage with the task (*n* = 1). The final sample included 60 children (22 males, 38 females) aged between 45 and 60 months (*M* = 53.22, s.d. = 3.84).

#### 3.1.2. Coding

Helping was coded per task on a scale of 0–4. If a child helped without any prompting, they received the highest score and the more prompts required the lower the helping score. Children who did not help at all received a score of 0. Coding is identical for this and all further studies in this manuscript. There were independent research assistants across the five studies who acted as the second coders. Full-coding scheme and reliability analyses for all studies are reported in the electronic supplementary material.

#### 3.1.3. Analytical approach

Generalized linear mixed models (GLMM) were used to account for potential non-independence of the data (trials nested within participants and varying testing locations). For all GLMMs, we used the most optimal random effects structure based on the lowest Akaike information criterion value. A Poisson GLMM was used to assess the effect of condition on the number of prompts before helping occurred. A chi-square analysis was used to assess whether conditions affected the occurrence of helping (yes or no), collapsed across trials. Finally, binomial GLMMs were used to establish whether conditions affected the tendency to console (yes or no) or disengage (yes or no) from the puppet for children who did not help only.

### 3.2. Results

In all studies, preliminary analyses revealed no difference between conditions in age and sex, suggesting random assignment was successful. Likewise, trial number and task type did not significantly affect helping behaviour, and these factors were not considered further. The datafiles and R script for each study is available on the Open Science Framework (https://osf.io/e78tb/?view_only=ff46e0f0a9894023b507af5a526afac6).

#### 3.2.1. Effect of cost on helping behaviour

**Figure 1.**
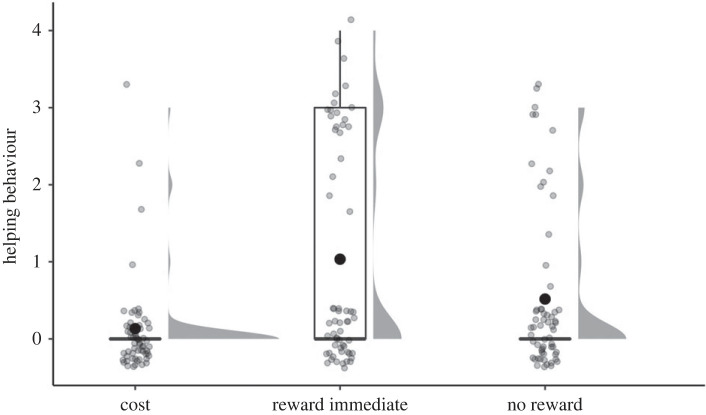
Boxplot of the effect of condition on helping behaviour, where higher values represent greater helping behaviour. Black dots represent means and grey dots represent individual datapoints (jittered for visualization purposes).

To assess the effect of condition on helping behaviour, a Poisson GLMM was used, with participant and location included as a nested random intercept. Results revealed a significant effect of condition, *χ*^2^(2, *N* = 180) = 8.53, *p* = 0.015 (figure 1). Follow-up Tukey contrasts revealed that helping behaviour was significantly higher for children in the reward immediate condition (*M* = 1.03, s.d. = 1.46) compared with children in the cost condition (*M* = 0.13, s.d. = 0.54; table 2). No other comparisons were significantly different.

**Table 2.** Statistical results for helping behaviour analysis by condition.

condition	estimate	s.e.	*p*
reward immediate versus cost	2.77	0.95	0.011
no reward versus cost	1.89	0.96	0.146
no reward versus reward immediate	−0.89	0.80	0.794

#### 3.2.2. Exploratory analysis

With our scoring system, children who helped immediately and consistently across tasks received a higher score than children who helped later and rarely. However, it is possible that proportionately more children helped in the conditions that involved a sticker as a reward (e.g. cost and reward immediate conditions) than those without a sticker reward (e.g. no reward). To examine this, we analysed whether the proportion of children helping differed across conditions. Additionally, for children who did not help, we examined whether the proportions of children who consoled or disengaged from the distressed puppet differed across conditions. These were coded in binary 0–1 (not helped/helped; not consoled/consoled; did not disengage/disengaged). See electronic supplementary material for full coding scheme.

##### 3.2.2.1. Helping

A total of 18/60 children helped the distressed puppet at least once across trials. This included 3/30 children in the cost condition, 8/20 children in the reward immediate condition and 7/20 children in the no reward condition. There was no significant difference in the proportion of children that helped across the three conditions based on a Chi-square analysis, *χ*^2^(2, *N* = 60) = 3.33, *p* = 0.189, *phi* = 0.236.

##### 3.2.2.2. Consoling

Children consoled the distressed puppet on 13/141 trials when they did not help. This included 6/56 trials in the cost condition, 2/39 trials in the reward immediate condition and 5/46 trials in the no reward condition. A binomial GLMM, with participant included as a random intercept, found no significant difference in rates of consoling when children did not help across all three conditions, *χ*^2^(2, *N* = 141) = 0.05, *p* = 0.974.

##### 3.2.2.3. Disengaging

Children disengaged from the distressed puppet on 64/141 trials when they did not help. This included 33/56 trials in the cost condition, 15/39 trials in the reward immediate condition and 16/46 trials in the no reward condition. A binomial GLMM, with participant included as a random intercept, found no significant difference in the proportion of children disengaging from the distressed puppet when they did not help across conditions, *χ*^2^(2, *N* = 141) = 3.82, *p* = 0.148.

In Study 1, we found that children help more in the immediate reward condition than the cost condition, indicating that cost does reduce helping. We also found no effect of condition on the proportion of children who helped, or the proportion of children who consoled or disengaged when they did not help.

## 4. Study 2: explicit instructions

In Study 2, we aimed to explore how explicit instructions impact children's compassionate responding. In the original paradigm [6], children were not given explicit instructions about whether they could help the puppet. In this way, the rules of the game were ambiguous, so children may have perceived that the compassionate action was against the rules and going against the rules is in itself a cost to children; moreover, items that we own are perceived as more costly to give up [30]. This aligns with findings that sharing behaviours rely on children's motivations *and* explicit cues for scaffolding and direction [31,32]. This carries important implications for observations of compassionate behaviours experimentally where children are in an unfamiliar situation with a stranger and may therefore require explicit instructions to overcome this inhibitor to compassionate responding. It may also be possible that children in Green *et al*. [6] interpreted the tasks as competitive (doing the same thing at the same time in a shared space, but not together). Competition has been shown to impede prosocial behaviour [33] and may also have acted as an inhibitor to compassion.

To examine how these two factors (instructions and perceived competition) influence compassionate responding, we again replicated the cost condition from the original paradigm. However, here we systematically varied whether children were given explicit instructions that they could share and the game was not a competition, or not (thus keeping the rules of the game ambiguous). We predicted that children who receive explicit permission to share would be more compassionate than those who do not.

### 4.1. Method

This study adapted the cost condition from the original paradigm [6] but varied whether the experimenter explicitly told children that they could share, demonstrated how to share prior to beginning the game and told children the game was not a competition (explicit instruction condition). This was in contrast with a condition where children were not given any additional information (implicit instruction condition, as per the original paradigm).

#### 4.1.1. Participants

In Study 2, 45 children were recruited, but five children were excluded from the final analysis due to participant or experimenter error. The final sample included 40 children (15 males, 25 females), aged between 48 and 60 months (*M* = 52.53, s.d. = 3.38).

### 4.2. Results

#### 4.2.1. Effect of instruction type on helping behaviour

To assess the effect of condition on helping behaviour, a Poisson GLMM was used, with participant included as a random intercept. Results revealed no significant effect of condition, *χ*^2^(1, *N* = 120) = 3.62, *p* = 0.057, such that there was no significant difference in helping behaviour in the explicit (*M* = 1.07, s.d. = 1.53) and non-explicit (*M* = 0.38, s.d. = 1.04) conditions.

#### 4.2.2. Exploratory analyses

##### 4.2.2.1. Helping

**Figure 2.**
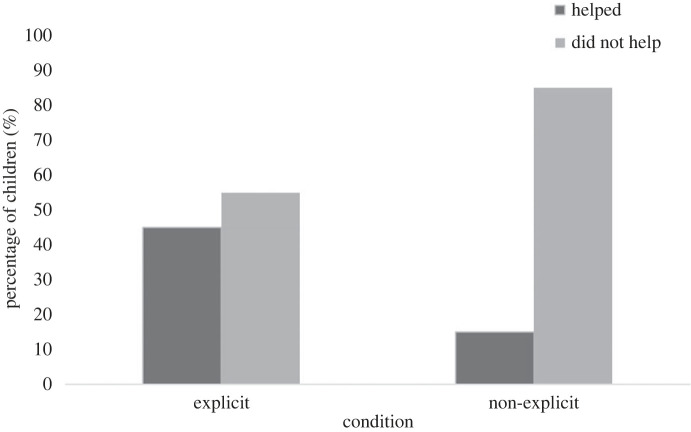
Percentage of children who helped or did not help within each condition.

A total of 12/40 children helped the distressed puppet at least once across trials. This included 9/20 children in the explicit instructions condition and 3/20 children in the non-explicit instructions condition (figure 2). A Chi-square analysis revealed the proportion of children who helped the puppet in the explicit instructions condition was significantly greater than the proportion of children who helped in the control condition, *χ*^2^(1, *N* = 40) = 4.29, *p* = 0.038.

##### 4.2.2.2. Consoling

Children consoled the distressed puppet on 2/91 trials when they did not help. This included 1/39 trials in the explicit condition and 1/52 trials in the non-explicit condition. A binomial GLMM, with participant included as a random intercept, found no significant difference in rates of consoling when children did not help across conditions, *χ*^2^(1, *N* = 91) = 0.03, *p* = 0.867.

##### 4.2.2.3. Disengaging

Children disengaged from the distressed puppet on 30/91 trials when they did not help. This included 14/39 trials in the explicit condition and 16/52 trials in the non-explicit condition. A binomial GLMM, with participant included as a random intercept, found no significant difference in rates of disengaging when children did not help across conditions, *χ*^2^(1, *N* = 91) = 0.24, *p* = 0.627.

In Study 2, we found that explicit instructions increased the proportion of children who helped, such that significantly more children helped the puppet in the explicit instruction condition than in the non-explicit instruction condition. We found no condition differences in overall rates of helping, or the proportion of children who disengaged or consoled the puppet when they did not help.

## 5. Study 3: not mine, but ours

In Study 3, we explore the role of resource ownership on children's compassionate responding. In the original paradigm [6], each child and puppet are given their own container of resources to complete the task. This may have created ownership bias—and past work shows that children are less likely to share resources that belong to them [25]. Svetlova *et al*. [25] theorize that this reflects children's inability to overcome possessiveness and object ownership to help another. Interestingly, children even display a preference for (endowment effect) items arbitrarily positioned as their own [34], defending these items aggressively compared with items ‘owned’ by the wider classroom [35].

Study 3 built on these findings in order to gain insight into how ownership effects motivations to act compassionately. We again replicated the cost condition from the Green *et al*. [6] paradigm but varied whether the resources were owned by the child (individual condition) or part of a shared resource pool (shared condition). We predicted that children would help more in the shared resource condition than the individual resource condition.

### 5.1. Method

This study adapted the cost condition from the original paradigm [6]. However, children's resources (e.g. puzzle pieces) were either presented in separate boxes, so the child had their own resources (individual condition, as per the original paradigm), or presented in a large communal box to indicate that they are from a shared resource pool (shared condition). In the shared condition, children were also explicitly told that the resources were shared.

#### 5.1.1. Participants

In Study 3, 46 children were recruited, but five children were excluded from the final analysis due to participant or experimenter error. The final sample included 41 children (20 males, 21 females), aged between 46 and 62 months (*M* = 53.07, s.d. = 4.18).

### 5.2. Results

#### 5.2.1. Effect of resource status on helping behaviour

To assess the effect of condition on helping behaviour, a Poisson GLMM was used, with participant included as a random intercept. Results revealed no significant effect of condition, *χ*^2^(1, *N* = 123) = 0.02, *p* = 0.881, such that there was no significant difference in helping behaviour between the shared resource (*M* = 0.63, s.d. = 1.21) and individual resource (*M* = 0.52, s.d. = 1.08) conditions.

#### 5.2.2. Exploratory analyses

##### 5.2.2.1. Helping

A total of nine children helped the distressed puppet at least once across trials. This included 5/21 children in the shared resources condition and 4/20 children in the individual resources condition. There was no significant difference in the proportion of children who helped across conditions based on a Chi-square test, *χ*^2^(1, *N* = 41) = 0.087, *p* = 0.768.

##### 5.2.2.2. Consoling

Children consoled the distressed puppet on 1/96 trials when they did not help. This included 1/48 trials in the individual resources condition and 0/48 trials in the shared resources condition. A binomial GLMM, with participant included as a random intercept, found no significant difference in rates of consoling when children did not help across conditions, *χ*^2^(1, *N* = 96) = 0.001, *p* = 0.974.

##### 5.2.2.3. Disengaging

Children disengaged from the distressed puppet on 64/96 trials when they did not help. This included 34/48 trials in the individual resources condition and 30/48 trials in the shared resources condition. A binomial GLMM, with participant included as a random intercept, found no significant difference in rates of disengaging when children did not help across conditions, *χ*^2^(1, *N* = 96) = 0.52, *p* = 0.469.

In Study 3, we found no effect of ownership on children's overall or proportionate helping, consoling or disengaging behaviour. Children were equally likely to help, and equally likely to disengage or console when they did not help, regardless of whether the task pieces were shared or owned by the individual.

## 6. Study 4: Who am I helping? (adult versus puppet target)

In Study 4, we aim to explore how the target of compassion influences children's compassionate responding. Across all variations of the original paradigm [6], puppets were used as the distressed target. Children possess the ability to attribute biological and psychological characteristics to inanimate objects including stuffed toys and imaginary companions [36]. Moreover, several studies have shown that children treat puppets in a similar manner to humans [37,38] and identify with a puppet in-group [39]. However, other work has found that children can understand human emotions better than puppet emotions [40]. Given that compassion is defined by the motivation to alleviate another's suffering, it is critical that children understand the suffering of the target. Perhaps children are not sufficiently sensitive to a puppet target to elicit compassionate responding. We, therefore, vary whether the target of compassion is a puppet or an adult human—predicting that children will behave more compassionately to human targets than puppet targets.

### 6.1. Method

We again replicated the cost condition in the original compassionate responding paradigm [6]. However, we varied whether the distressed agent was a puppet (puppet condition) or an adult human (adult condition). Unlike other conditions, in the adult condition, we also had two adult experimenters, one who was the confederate in the study completing the tasks with the child, and a second adult experimenter providing instructions. We therefore measured social referencing behaviour of the children to the adult experimenter.

#### 6.1.1. Participants

In Study 4, 44 children were recruited, but four children were excluded from the final analysis due to participant or experimenter error. The final sample was 40 children (14 males, 26 females), aged between 47 and 60 months (*M* = 54.15, s.d. = 3.40).

### 6.2. Results

#### 6.2.1. Effect of agent type on helping behaviour

To assess the effect of condition on helping behaviour, a Poisson GLMM was used, with participant included as a random intercept. Results revealed no significant effect of condition, *χ*^2^(1, *N* = 120) = 1.13, *p* = 0.288, such that there was no significant difference in helping behaviour between the adult (*M* = 0.22, s.d. = 0.69) and puppet (*M* = 0.50, s.d. = 1.03) conditions.

#### 6.2.2. Exploratory analyses

##### 6.2.2.1. Helping

A total of 11 children helped the distressed agent at least once across trials. This included 4/20 children in the adult condition and 7/20 children in the puppet condition. A Chi-square analysis revealed the proportion of children helping the agent did not significantly differ by condition, *χ*^2^(1, *N* = 40) = 1.13, *p* = 0.288.

##### 6.2.2.2. Consoling

Children consoled the distressed agent on 5/99 trials when they did not help. This included 2/53 trials in the adult condition and 3/46 trials in the puppet condition. A binomial GLMM, with participant included as a random intercept, found no significant difference in rates of consoling when children did not help across conditions, *χ*^2^(1, *N* = 99) = 0.02, *p* = 0.887.

##### 6.2.2.3. Disengaging

Children disengaged from the distressed agent on 40/99 trials when they did not help. This included 21/53 trials in the adult condition and 27/46 trials in the puppet condition. A binomial GLMM, with participant included as a random intercept, found no significant difference in rates of disengaging when children did not help across conditions, *χ*^2^(1, *N* = 99) = 0.01, *p* = 0.937.

##### 6.2.2.4. Social referencing

In the adult condition, when the adult became distressed, children demonstrated social referencing on 34/60 trials by looking at the other experimenter in the room. In trial 1, 12/20 children socially referenced. In trials 2 and 3, 11/20 children socially referenced. A binomial GLMM, with participant included as a random intercept, found no significant difference in whether children helped on a trial, based on whether they socially referenced or not, *χ*^2^(1, *N* = 60) = 2.25, *p* = 0.133.

In Study 4, we found no effect of recipient on children's helping, such that they helped adults and puppets the same amount, were equally likely to help adult and puppets, and equally likely to console and disengage from adults and puppets when they did not help. This finding could have been partly driven by having a third party, the adult experimenter in the room, who the child often referred to as a possible gauge on what to do.

## 7. Study 5: help me, we are in the same group!

In Study 5, we test a different social boundary of compassion: group membership. There is extensive literature demonstrating that young children are strongly biased toward in-groups. Children like in-group members more [21,39,41,42] prefer them as social learning models for both selective trust [43] and imitation tasks [43,44], and also behave more prosocially towards them; opting to share resources with them over others [45]. Yet, no work has examined group biases in the context of explicit compassion. Interestingly, our sense of group membership is malleable and can be influenced by contextual factors. Research using minimal group paradigms, where group membership is arbitrary (i.e. temporarily assigning group colour), found that it is sufficient to induce in-group preference and helping [21,46–49]. We, thus, examined how group bias in a minimal group context would influence compassionate behaviour. We predicted that children would be more compassionate to those who are group members compared with those who are not.

### 7.1. Method

We again replicated the cost condition in the original compassionate responding paradigm [6]. However, we varied whether the recipient of help (the puppet) was a member of the child's group or not. There were three levels of group membership: no group, group assignment and enhanced group assignment. In the group assignment conditions, a minimal group paradigm was used where the child and puppet were assigned to the same group, which was established by them wearing the same group colour (a yellow sash and bandana). Children and puppets were assigned to groups by drawing out a coloured token from the same drawstring bag. However, unbeknown to children, there were only yellow tokens and thus, children and puppets were always in the same yellow group. The group enhancement condition also included the puppets verbally highlighting their shared group membership with the children. This was done by including an additional sentence to the original distress prompts (see electronic supplementary material). Thus, children were either assigned to a no group condition, to the same group as the puppet using a minimal group paradigm (group assignment condition), or to the same group as the puppet and the puppet referenced this in their distress (e.g. ‘*We're in the same group, you are getting a sticker but I won't’*) (group enhancement condition).

#### 7.1.1. Participants

In Study 5, a total of 82 children participated in this experiment. Five children were excluded from the final analysis due to experimenter error or participant error. The final sample included 77 children (35 males: 42 females) aged between 44 and 61 months (*M* = 53.21, s.d. = 3.92).

### 7.2. Results

#### 7.2.1. Effect of group membership on helping behaviour

To assess the effect of condition on helping behaviour, a Poisson GLMM was used, with participant included as a random intercept. Results revealed no significant effect of condition, *χ*^2^(2, *N* = 231) = 1.96, *p* = 0.375, such that there was no significant difference in helping behaviour between the no group (*M* = 1.08, s.d. = 1.39), group assignment (*M* = 0.76, s.d. = 1.24) and group enhancement (*M* = 0.71, s.d. = 1.33) conditions.

#### 7.2.2. Exploratory analyses

##### 7.2.2.1. Helping

A total of 33/77 children helped the puppet when it was distressed at least once across trials: 12/25 children in the no group condition, 14/28 in the group assignment condition and 7/24 in the group enhancement condition. There was no significant difference in the proportion of children that helped the puppet across all the conditions, *χ*^2^(2, *N* = 77) = 2.69, *p* = 0.261.

##### 7.2.2.2. Consoling

Children consoled the distressed puppet on 24/160 trials when they did not help. This included 5/45 trials in the no group condition, 9/60 trials in the group assignment condition and 10/55 trials in the group enhancement condition. A binomial GLMM, with participant included as a random intercept, found no significant difference in rates of consoling when children did not help across conditions, *χ*^2^(2, *N* = 160) = 0.16, *p* = 0.922.

##### 7.2.2.3. Disengaging

Children disengaged from the distressed puppet on 77/160 trials when they did not help. This included 17/45 trials in the no group condition, 28/60 trials in the group assignment condition and 32/55 trials in the group enhancement condition. A binomial GLMM, with participant and location included as a nested random intercept, found no significant difference in rates of disengaging when children did not help across conditions, *χ*^2^(2, *N* = 160) = 2.43, *p* = 0.297. We do not find any effect of group membership on overall rates of helping, or the proportion of children who engaged in helping, consoling or disengaging behaviour when they did not help.

## 8. General discussion

In this research, we aimed to explore the bounds of compassion in young children. Across five studies, we examined the role of cost (Studies 1–3) and the recipient of compassion (Studies 4 and 5) in 4- to 5-year-old children using an adapted version of an established compassionate responding paradigm [6]. We found strong evidence that cost reduces compassionate responding. When children were able to help without sacrificing their reward, rates of helping increased. Similarly, when they were given explicit instructions indicating that they could help—and thus helping was foregrounded—rates increased. By contrast, the recipient of compassion did not appear to influence compassionate responding: children were equally likely to help a human adult and a puppet, and an in-group member and neutral agent.

These findings indicate that personal cost is a greater inhibitor to compassionate responding than who compassion is directed toward. This aligns with adult literature which suggests that personal cost to self [16] and competitive self-interest [17] are inhibitors to compassion. Loewenstein & Small [8] proposed a prosocial model suggesting that when we engage in compassionate actions we are either moved emotionally or we cognitively deliberate on whether to take compassionate action. In our series of studies, it could be that children are deliberating on the costs and benefits of acting compassionately, with the cost of forgoing the reward being greater than anticipated benefits to the target. This may be specific to this age group, with older children perceiving more accurately the benefit compassionate actions have for the target. It could also be that for the 4-year-olds in the current studies the cognitive control to forgo own rewards for the benefit of others is too costly, and compassionate helping is avoided. However, not all methods of reducing cost increased compassion. In Study 3, drawing from a pool of shared resources had little impact on how children perceived cost and responded to suffering from the puppet. It is possible that children saw the ‘finite’ amount of resources available in the shared bucket and realized they had to get the pieces they needed before the puppet. In this way, the shared resource condition may have primed competitive self-interest, as opposed to increasing compassionate behaviour.

### 8.1. Limitations and future directions

Although rates of helping increased when cost was removed or reduced, rates were still low overall; none of our manipulations led to better than moderate rates of helping. Even in the explicit instruction condition only 50% of the children helped, and they only helped after a series of distress prompts. Although this is consistent with past research detailing that young children are often unwilling to engage in costly prosocial behaviour [50], it is possible that something about this particular paradigm produces low helping rates. For example, the parallel nature of both puppet and child completing the same task may have led to a more competitive environment. A different procedural paradigm where the child and puppet are completing different tasks or activities that still manipulates cost and distress might be able to shed further light into this compassionate responding. Additionally, participants' cultural backgrounds may have contributed to their low-level helping. Past work has raised the possibility of cross-cultural differences arising in helping behaviour [51–53]. What we do not know is the extent to which contrasting cultural perspectives (e.g. between those with an individualistic versus collectivist outlook) might result in different responding on tasks such as those presented here. This remains a topic for future exploration.

In Study 4, the target (puppet or adult) did not appear to greatly influence the child's compassionate responding. This suggests that children may be, at least somewhat, impartial in their compassionate responding. This contrasts the dominant narrative that young children are self-serving [54], but aligns with recent work suggesting that children may be more morally expansive than adults in certain contexts [55].

There are also other facilitators that have been shown to influence compassionate responding in adults that were not tested here, such as prosocial modelling, or perhaps a target where a bond has been formed, or where the puppet is a liked or disliked target. Notably, most of these suggested factors vary at an individual level, rather than being context specific as was the case for all facilitators and inhibitors tested here. As such, these individual differences may represent a more promising avenue to increasing compassionate responding. A developmental analysis was not explored in the current studies, and older children may respond differently. As children age, social norms are more embedded and children display greater self-control, thus children from this age may be more willing (or able) to engage in compassionate behaviour, as has been found with other prosocial and altruistic behaviours [55–57]. Expanding on this, the research of compassion science could benefit greatly by designing experiments that have clear cost and benefit dilemmas included. This would lead to more ecologically valid paradigms, as in everyday life people are constantly having to deliberate the pros and cons of acting compassionately. Currently, most prosocial research with children does not include clear costs, and while there is some work examining costs to prosocial helping in adolescents [58,59] and adulthood [60], continued systematic examination of the cognitively deliberative task of cost-benefit decisions to acting compassionately is needed to understand the complex prosocial construct of compassion. Finally, across our studies, children were able to infer the distressed emotional state of the target accurately, but we did not ask whether the child acted to assuage the target's (puppet/adult) distress. If children did not help or console the target, they frequently disengaged from them (e.g. looking away or changing the topic of conversation). The disengagement may further indicate that children realized the emotional distress of the target and were attempting to avoid negative experiences, such as guilt or shame, for not helping them. Qualitative work asking children (i) why they helped and (ii) how they felt when not helping, could provide further insights into how children understand the intent of their prosocial actions and their consequences.

## 9. Conclusion

We provide evidence of the bounds of children's compassionate responding in a behavioural task. We find strong evidence that personal cost is a key inhibitor to compassionate behaviour, which in turn suggests that reducing cost may facilitate compassion. Our findings also indicate that young children are impartial in where their compassion is directed—being similarly willing to help in-groups and out-groups, as well as humans and puppets. Finally, we find behavioural evidence of guilt or shame associated with not helping. Taken together, these five studies provide a systematic exploration of contextual factors that inhibit and facilitate compassionate behaviour in young children.

## Data Availability

Available on the Open Science Framework (https://osf.io/e78tb/?view_only=ff46e0f0a9894023b507af5a526afac6). The data are provided in the electronic supplementary material [61].
